# Recent advances in understanding the roles of hypocretin/orexin in arousal, affect, and motivation

**DOI:** 10.12688/f1000research.15097.1

**Published:** 2018-09-06

**Authors:** Natalie Nevárez, Luis de Lecea

**Affiliations:** 1 Department of Psychiatry & Behavioral Sciences, Stanford University, Stanford, California, USA

**Keywords:** hypothalamus, vigilance, arousal, wake, sleep, addiction, memory

## Abstract

The hypocretins (Hcrts) are two alternatively spliced neuropeptides (Hcrt1/Ox-A and Hcrt2/Ox-B) that are synthesized exclusively in the hypothalamus. Data collected in the 20 years since their discovery have supported the view that the Hcrts play a broad role in the control of arousal with a particularly important role in the maintenance of wakefulness and sleep-to-wake transitions. While this latter point has received an overwhelming amount of research attention, a growing literature has begun to broaden our understanding of the many diverse roles that the Hcrts play in physiology and behavior. Here, we review recent advances in the neurobiology of Hcrt in three sections. We begin by surveying findings on Hcrt function within normal sleep/wake states as well as situations of aberrant sleep (that is, narcolepsy). In the second section, we discuss research establishing a role for Hcrt in mood and affect (that is, anxiety, stress, and motivation). Finally, in the third section, we briefly discuss future directions for the field and place an emphasis on analytical modeling of Hcrt neural activity. We hope that the data discussed here provide a broad overview of recent progress in the field and make clear the diversity of roles played by these neuromodulators.

## Introduction

In 1998, two research studies published within a month of each other described a set of novel hypothalamic peptides. The first group to describe them was led by Gregor Sutcliffe at the Scripps Research Institute in La Jolla, California. The Sutcliffe group used subtractive RNA hybridization to characterize a cDNA clone with restricted expression in the dorsal and lateral hypothalamus (LH). This cDNA clone encoded a preproprotein termed preprohypocretin. This was the putative precursor to two peptides that they named hypocretin-1 (Hcrt1/Ox-A) and hypocretin-2 (Hcrt2/Ox-B) with respective receptors OX _1_R and OX _2_R. Their name was a combination of *hypo* for their hypothalamic origin and *cretin* based on their sequence homology to the gut hormone secretin ^1^. At the same time, Masashi Yanagisawa’s group at University of Texas Southwestern was characterizing ligands for orphan G-protein-coupled receptors as a means to determine their role in various physiological processes. The group found two extracts within the hypothalamus that bound and activated two orphan receptors with unknown functions. When supraphysiological doses of peptide were injected intracerebroventricularly, these peptides promoted food intake. Owing to this effect, the group named the peptides “orexins” based on the Greek word for appetite ( *orexis*) ^2^. Indeed, the two groups were describing the same peptides, and today hypocretin and orexin are synonymous. Here, we will review some of the most recent findings in the neurobiology of Hcrt in relation to arousal, emotional processing, and motivation and finally discuss future directions for analytical modeling of Hcrt networks.

As new tools have become increasingly accessible to researchers at all levels, we have seen an explosion of studies using specific methodologies for the study of neural circuitry, namely the use of optogenetics and chemogenetics for the manipulation of neural circuits, fiber photometry and microendoscopy for the measurement of cellular activity via genetically encoded calcium indicators (for example, GCaMP6f), and precise genetic tools (for example, transcription activator-like effector nucleases [TALENs]; targeting-induced local lesions in genomes [TILLING]; and clustered regularly interspaced short palindromic repeats [CRISPR/Cas9]) and high-throughput sequencing to characterize and manipulate genes. Optogenetics is a technique in which neurons are genetically modified to express light-sensitive ion channels (for example, channelrhodopsins and archaerhodopsins). Subsequent photostimulation of these neurons can activate or inhibit cells on the basis of the wavelength and intensity of light used ^3^. Chemogenetics uses modified G-protein-coupled receptors (designer receptors exclusively activated by designer drugs, also known as DREADDs) that are largely activated by a metabolite of clozapine N-oxide (CNO) when injected systemically ^4^. Excitatory or inhibitory DREADDs can be selectively expressed in neuronal populations of interest (for example, in a Cre- or Flp-dependent manner) which then can be manipulated by injections of CNO ^5^. Additionally, the expression of calcium indicators allows the measurement of cell activity in relation to behavior via fiber photometry or microendoscopy ^6^. Most recently, genome editing via CRISPR/Cas9 systems and developmental engineering can quickly produce knock-outs or knock-ins for multiple gene targets in a single generation ^7– 10^. As our review focuses primarily on advances made within the past 3 years, there is an overwhelming representation of these methodologies, which already have significantly advanced our understanding of the Hcrt circuit ^7, 11, 12^.

## Part I: hypocretin and arousal

Hcrt cell bodies reside exclusively within the hypothalamus and project broadly throughout the brain and spinal cord ^13^. They receive major inputs from a diversity of afferents covering all of the major neurotransmitter systems ^14^. The increasing database of research on Hcrt shows that these neuropeptides may not be necessary for the generation of sleep or wakefulness per se but rather for coordinating and stabilizing these states. Hcrt activity regulates sleep-to-wake transitions via its many interactions with other neuroanatomical and neurotransmitter systems ^15, 16^. Thus, many of the recent findings discussed here are a combination of studies done directly on Hcrt circuitry or studies done on other systems that either coordinate activity with or are modulated by Hcrt.

### Sleep and wakefulness

Hcrt deficiency underlies the majority of cases of narcolepsy ^17– 20^. Narcolepsy is characterized by unexpected sleep episodes during times of wakefulness, excessive daytime sleepiness, rapid eye movement (REM)-like episodes that can co-occur with conscious wakefulness, and disrupted nocturnal sleep ^21, 22^. Further support for aberrant state boundaries in narcolepsy was recently published showing intrusions of REM sleep during wakefulness as well as intrusions of non-REM (NREM) sleep during wakefulness ^23^. While it is established that Hcrt neuron degeneration contributes to the etiology of narcolepsy in many cases, recent evidence has characterized how sleep and wakefulness are impacted through the progression of Hcrt cell loss ^17, 18, 24^. Studies in mice at different stages of Hcrt neuron degeneration found that loss of these neurons reduces the likelihood of long wake bouts but increases the likelihood of short wake bouts (that is, wakefulness is fragmented) as a result of waking primarily during the first 30 seconds of NREM sleep and a reduced likelihood of returning to sleep within the first 60 seconds of wakefulness ^24^. **

**Figure 1.**
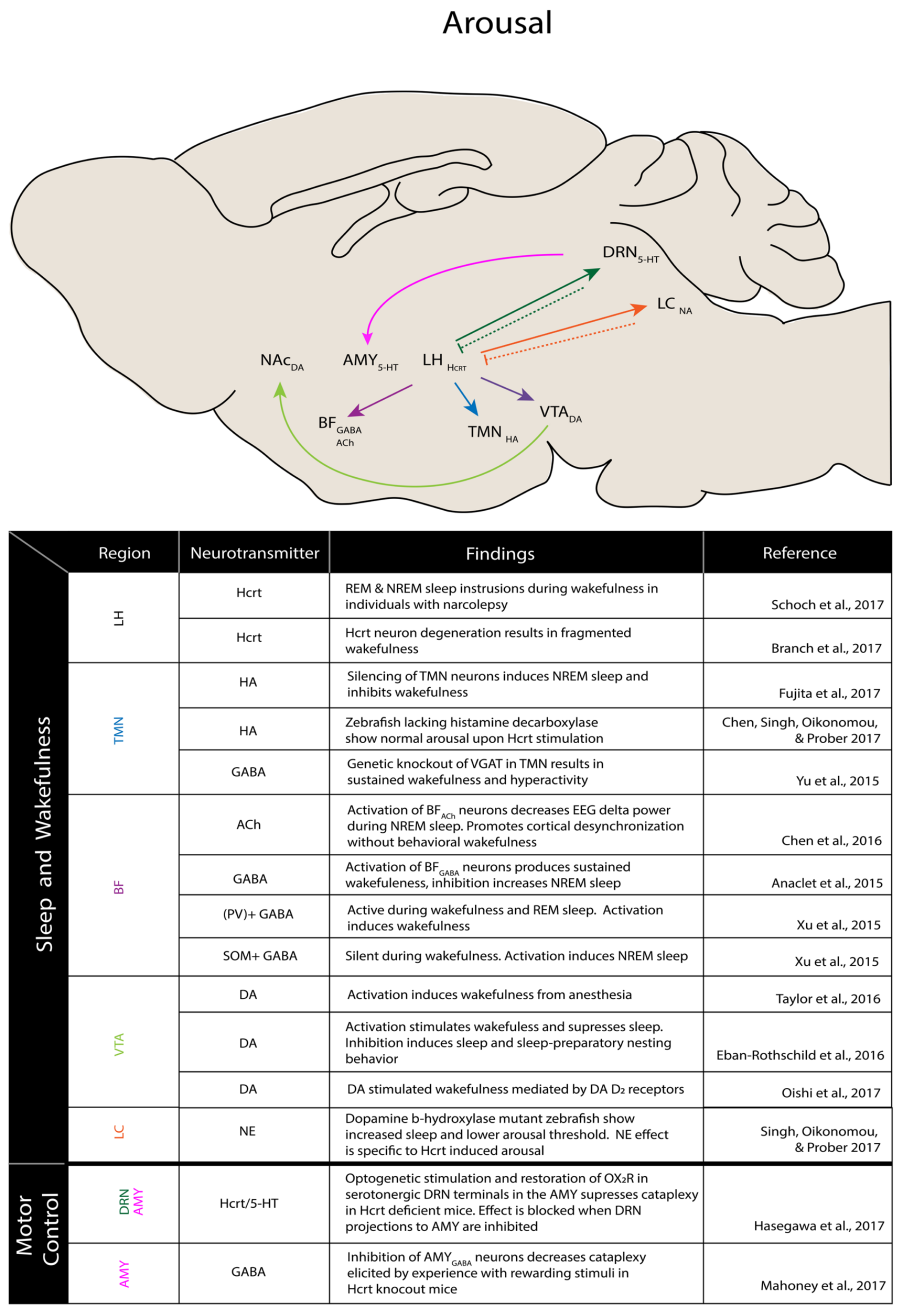
Hypocretin arousal network. Research of the past three years has found evidence of hypocretin-associated arousal in the illustrated circuits. Solid lines denote excitatory projections, and dashed lines denote inhibitory projections. 5-HT, serotonin; ACh, acetylcholine; AMY, amygdala; BF, basal forebrain; DA, dopamine; DRN, dorsal raphe nucleus; GABA, gamma aminobutyric acid; HA, histamine; Hcrt, hypocretin; LC, locus coeruleus; LH, lateral hypothalamus; NA, noradrenergic system; NAc, nucleus accumbens; NE, norepinephrine; NREM, non-rapid eye movement; PV, parvalbumin; REM, rapid eye movement; SOM, somatostatin; TMN, tuberomammillary nucleus; VTA, ventral tegmental area.

While early observations demonstrated that Hcrt deficiency underlies narcolepsy, a causal role for Hcrt in sleep-to-wake transitions was shown only in 2007 ^25^. Optogenetic manipulations of Hcrt circuitry revealed that activation of this neuronal population induces wakefulness in mice while optogenetic inhibition promotes NREM sleep ^25, 26^. Likewise, chemogenetic studies targeting Hcrt neural activity have shown that injections of CNO in mice expressing excitatory (Gq) DREADDs promote wakefulness but that engagement of inhibitory (Gi) DREADDs decreases wakefulness and increases time in NREM sleep ^27^. Thus, Hcrt clearly plays a critical role in the regulation of sleep-to-wake transitions, but its various effects on these processes are regulated by the many brain regions and neurotransmitter systems with which it interacts. Indeed, research has demonstrated important interactions between Hcrt and histaminergic neurons within the tuberomammillary nucleus (TMN), cholinergic and GABAergic neurons of the basal forebrain (BF), dopamine (DA) neurons within the ventral tegmental area (VTA), and norepinephrine (NE) neurons of the locus coeruleus (LC), among others ^28^ ( Figure 1). Recent advances in our understanding of the roles of these regions in sleep/wake regulation and their possible interactions with the Hcrt system are outlined below.

As we discuss below, histaminergic neurons of the TMN play a role in arousal, but the ways in which Hcrt influences TMN-mediated arousal are not clear. TMN histaminergic neurons become active during wake onset and are silent during sleep ^29, 30^. Optogenetic silencing of histaminergic TMN neurons induces NREM sleep and inhibits wakefulness ^31^. Hcrt activates TMN neurons and increases histamine release at their terminals, suggesting that Hcrt activation of TMN neurons supports wakefulness ^32– 34^. However, mice and zebrafish that lack the rate-limiting enzyme in histamine synthesis (histamine decarboxylase) show normal sleep-to-wake transitions upon optogenetic stimulation of Hcrt neurons ^35, 36^. These data suggest that histaminergic signaling in the TMN may serve a redundant function in Hcrt-mediated arousal. Recent findings also show that histaminergic regulation of wakefulness within the TMN may be via co-transmission of GABA. Small interfering RNA (siRNA)-mediated knockdown of the vesicular GABA transporter (VGAT) or genetic knockout of the VGAT gene in histaminergic neurons results in hyperactivity and sustained wakefulness ^37^. Future studies should characterize how manipulations of GABA transmission in the TMN impacts Hcrt-induced wakefulness specifically.

The BF is an attention- and arousal-sustaining structure containing cholinergic, GABAergic, and glutamatergic cells that are depolarized by Hcrt ^38^. Similarly, the region expresses both Hcrt receptors, and there is a higher density of OX _2_R than OX _1_R ^39^. This difference may be meaningful, as studies in organotypic slice cultures show that Hcrt depolarizes cholinergic cells of the BF via actions at OX _2_R but not OX _1_R ^38^. However, injections of Ox-A into the BF of rats resulted in wakefulness in regions of the BF that show stronger expression of OX _1_R ^40^. Chemogenetic studies demonstrate that activation of cholinergic neurons of the BF decreases electroencephalogram (EEG) delta power (specifically during NREM sleep) and promotes cortical desynchronization without behavioral wakefulness ^41^. In contrast, activation of GABAergic neurons in this region produces sustained wakefulness whereas inhibition increases NREM sleep ^42^. Further genetic targeting studies show that subsets of GABAergic neurons in the region exhibit a diversity of responses across arousal states ^43– 45^. For example, parvalbumin-positive (PV ^+^) GABAergic neurons are more active during wakefulness and REM sleep than during NREM sleep whereas somatostatin-positive (SOM ^+^) GABAergic neurons are reciprocally silent during wakefulness. Predictably, optogenetic activation of PV ^+^ GABA neurons powerfully induces wakefulness whereas activation of SOM ^+^ GABAergic neurons promotes NREM sleep ^46– 49^. Modern genetic tools will continue to allow more detailed examinations of the impact of neuronal heterogeneity within regions in the context of Hcrt-mediated arousal.

The BF receives projections from midbrain DA neurons which may underlie the coupling of motivation to arousal states. Indeed, Hcrt axons project to midbrain DA neurons, and DA cell bodies express Hcrt receptors ^13, 50, 51^. *In vitro* electrophysiological recordings show that Hcrt1 and Hcrt2 treatment increases VTA DA neural firing ^52^. Hcrt1 injections into the VTA increase time awake and levels of DA at axonal terminals in the prefrontal cortex ^53, 54^. Although Hcrt neurons project to systems for all the monoamines and drugs that increase DA transmission increase wakefulness, DA was thought not to be involved in normal sleep/wake regulation until recently ^55– 60^. Work from our laboratory has shown a role for VTA DA neurons in promoting arousal and the initiation of sleep-preparatory behaviors ^61^. Optogenetic activation of VTA DA neurons induces emergence from anesthesia, and chemogenetic activation of the VTA induces and consolidates wakefulness ^62, 63^. Further manipulations have demonstrated that VTA effects on wakefulness are through a D _2_ receptor-mediated mechanism ^62, 63^. Future work using projection-specific manipulations of Hcrt fibers within the VTA should better characterize their role in VTA-mediated arousal.

Noradrenergic neurons of the LC are strong promoters of arousal ^64, 65^. Direct administration of Hcrt1 into the LC increases firing rates while optogenetic silencing of these neurons with concurrent excitation of Hcrt cells prevents Hcrt-evoked sleep-to-wake transitions ^66– 68^. Additional studies have shown that noradrenergic activity is required to promote wakefulness and Hcrt-induced arousal in zebrafish. Using DA b-hydroxylase (dbh) (the rate-limiting enzyme in NE synthesis) mutant zebrafish, researchers found that these animals had dramatically increased sleep yet lower arousal thresholds ^69^. Additionally, wakefulness induced by genetic overexpression of Hcrt and optogenetic activation of Hcrt neurons is blocked by the inhibition or knocking out of NE in zebrafish larvae ^69^. However, further investigations have shown that overexpression of Hcrt or activation of Hcrt neurons has no significant effect in dbh mutant zebrafish ^35^. Thus, future work should continue to parse out the roles in which NE functions in sleep/wake regulation and how it may serve specifically within the Hcrt circuit to help regulate wakefulness in particular.

### Motor tone

Despite evidence demonstrating innervation of motor control systems by the Hcrt neurons, the coupling of arousal states with motor control is poorly understood ^70^. Indeed, measures of muscle tone along with cortical activity are the most common endpoints for characterizing various arousal states. A hallmark of waking is low-amplitude, high-frequency EEG activity with high muscle activity. REM sleep, also known as paradoxical sleep, is characterized by a near complete loss of skeletal muscle activity and an EEG resembling wakefulness. Hcrt-deficient narcoleptics show cataplexy (a loss of muscle tone during wakefulness that can result in postural collapse and can be triggered by strong emotions such as happiness and fear) ^22, 71– 74^. Similarly, individuals with REM sleep behavior disorder (RBD) show muscle tone problems. Under normal conditions, REM sleep is devoid of skeletal muscle tone; however, in RBD, an individual acts out their dreams by moving their limbs or talking, which can be dangerous for the individual enacting their dreams as well as anyone in their surroundings ^75^. Noradrenergic activity is necessary for motor behavior ^76^. Indeed, NE depletion has been shown to have a stronger motor-impairing effect than dopaminergic lesions with MPTP (1-methyl-4-phenyl-1,2,3,6-tetrahydropyridine) infusions of NE-induced hyperactivity, and loss of NE neurons is associated with motor learning deficits in aged rats ^77– 80^. Likewise, increasing noradrenergic tone has been shown to reduce cataplectic episodes ^81^. As discussed above, noradrenergic neurons of the LC are powerfully regulated by Hcrt; Hcrt dysfunction predictably alters both arousal and motor control. Moreover, Hcrt neurons project to dorsal raphe nucleus (DRN) serotonergic neurons where they may further influence motor behavior. Indeed, restoration of OX _2_R into serotonergic DRN neurons of dual Hcrt receptor knockout mice suppresses cataplexy-like episodes yet has no effect on sleep/wake fragmentation. Likewise, optogenetic stimulation of serotonergic DRN terminals in the amygdala (AMY) suppresses cataplexy-like arrests in Hcrt-deficient mice, and optogenetic inhibition blocks the cataplexy-reducing effect of Hcrt receptor restoration in serotonergic DRN neurons ^82^. Additional chemogenetic manipulations of this amygdalar circuit show that GABAergic populations of the central AMY are responsible for the production of cataplexy in mice but may not be the only circuit that can drive emotionally driven cataplexy ^83^. Together, these findings demonstrate a key role for amygdalar circuits in the production of cataplexy; however, they do not rule out other nuclei or circuits that may influence emotionally driven cataplexy. Indeed, the neural infrastructure exists for Hcrt activity to modulate AMY activity via its connections from the LC and DRN, and future studies should characterize the influence of Hcrt in emotion-driven cataplexy. **

## Part II: affect and motivation

**Figure 2.**
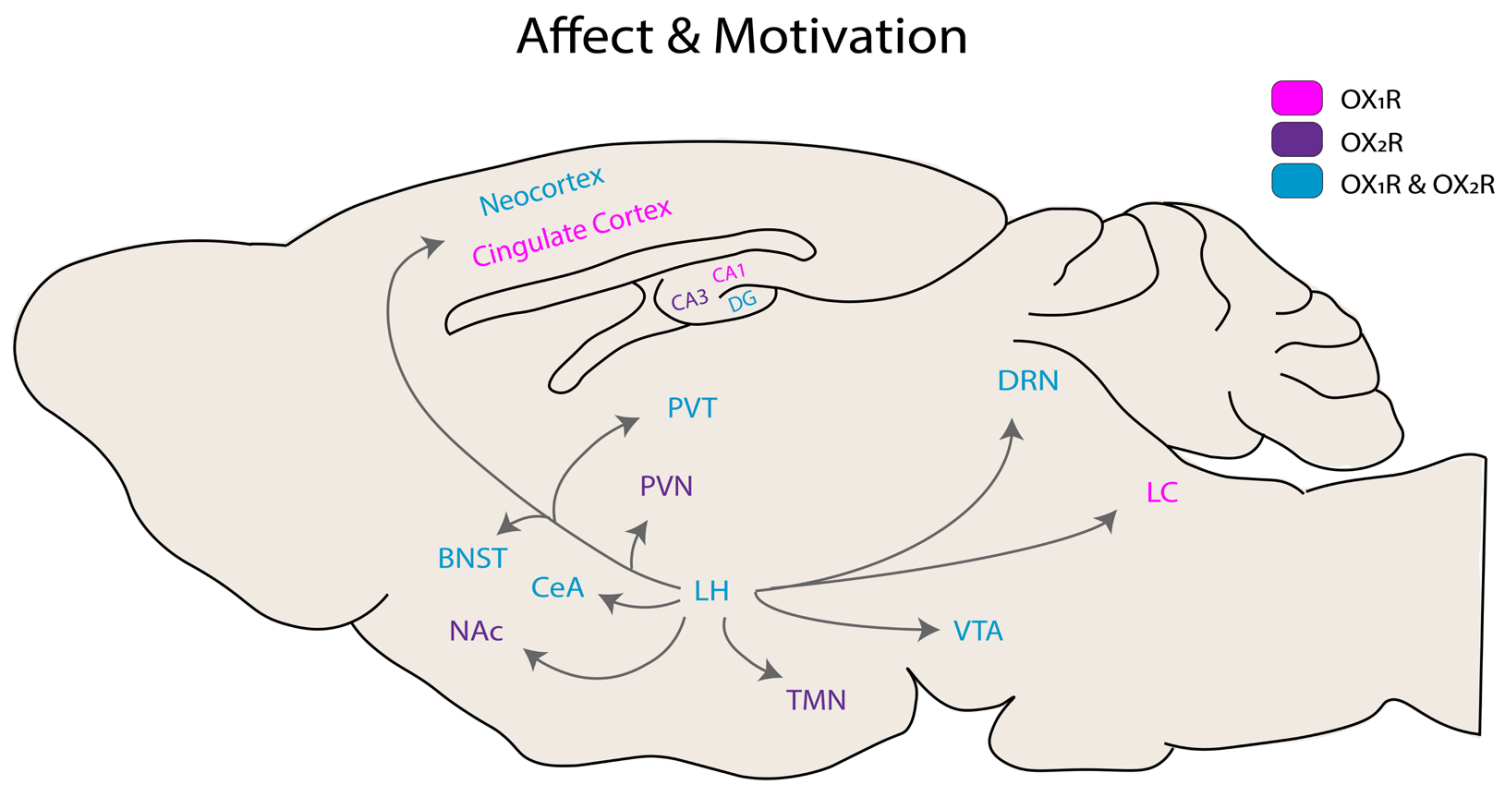
Hypocretin receptor distribution in the rodent brain. BNST, bed nucleus of the stria terminalis; CeA, central amygdala; DG, dentate gyrus; DRN, dorsal raphe nucleus; LC, locus coeruleus; LH, lateral hypothalamus; NAc, nucleus accumbens; PVN, paraventricular nucleus; PVT, paraventricular nucleus of the thalamus; TMN, tuberomammillary nucleus; VTA, ventral tegmental area.

As a regulator of arousal, the Hcrt system plays additional important roles in adaptive behaviors such as the regulation of stress responses and the avoidance of punishments and seeking of rewards. Additionally, sleep supports the consolidation of memory; predictably, proper regulation of sleep and arousal is key to proper memory function. Below we discuss recent findings in the growing field of Hcrt in the regulation of emotion and motivation and place a particular focus on stress and anxiety, addiction, and memory processes. Many of the data discussed here were gathered via global manipulations of Hcrt receptor signaling and thus should be interpreted in the context of known receptor distributions, drug treatments and selectivity (as many of these drugs are known to vary in selectivity on the basis of dose ^84^), and drug administration schedules ( Figure 2 and Table 1).

**Table 1.** Summary of recent findings for hypocretin in relation to affect and motivation. Colors match receptor representation in Figure 2: pink, OX _1_R manipulation; purple, OX _2_R manipulation; blue, OX _1_R/OX _2_R manipulation. AMY, amygdala; CO _2_, carbon dioxide; CPP, conditioned place preference; DA, dopamine; DG, dentate gyrus; EtOH, ethanol; Hcrt, hypocretin; LH, lateral hypothalamus; PeF OX, perifornical area orexin; PVT, paraventricular nucleus of the thalamus; VTA, ventral tegmental area.

		Manipulation	Findings	Reference
Stress and Anxiety		Compound 56	Subcutaneous treatment attenuated panic behaviors in 2 models of panic vulnerability (PeF OX disinhibition and sodium lactate treatment). No effect on sleep duration	Bonaventure *et al*., 2015
		JNJ-54717793	Attenuation of panic behavior and cardiovascular response in sodium lactate and CO _2_ panic models	Bonaventure *et al*., 2017
		Compound 56 SB-334867	Attenuation of CO _2_ induced anxiety and cardiovascular responses. No apparent sedative effects	Johnson *et al*., 2015
		SB-334867	Reduction in orofacial pain associated anxiety	Bahaaddini, Khatamsaz, Esmaeili-Mahani, Abbasnejad, & Raoof, 2016
		SB-334867	Effect on one measure of arousal (mobility in open field) in adolescent males. No effect on anxiety related behavior	Blume, Nam, Luz, Bangasser, & Bhatnagar, 2018
		OX _1_R Knockout	Increased anxiety, reduced social interaction, increased startle	Abbas *et al.*, 2015
		SORA2 JNJ- 10397049	No effect on anxiety or cardiovascular responses to CO _2_ model of panic induction	Johnson *et al.*, 2015
		DORA-12	Attenuation of CO _2_ induced anxiety responses.	Johnson *et al.*, 2015
		OX Knockout	Increased anxiety in open field, predator scent, and light/dark box	Khalil & Fendt, 2017
Motivation and Addiction	Cocaine	SB-334867	Blocks cue induced reinstatement with strongest effect in animals with highest cocaine-cue dependent behavior	Bentzley & Aston-Jones, 2015
		SB-334867	Decreased cocaine self administration and reduced cellular response to drug	Prince, Rau, Yorgason, & España, 2015
		RTIOX-276	Reduced responding for cocaine under high effort conditions, reduced DA response to cocaine paired cues	Levy *et al.*, 2017
		VTA OX _1_R Knockdown	Delays acquisition of self-administration, reduces response to drug under progressive ratio, alters DA transmission in striatum	Bernstein, Badve, Barson, Bass & España, 2017
		4PT	No effect on cocaine self administration or DA respone to drug	Prince, Rau, Yorgason, & España, 2015
		Almorexant	Reduced self administration under progressive ratio. Differential effects on DA response to drug over time	Prince, Rau, Yorgason, & España, 2015
		Suvorexant	Reduces self-administration under progressive ratio, cocaine induced ultrasonic vocalizations, and conditioned place preference. Reduces DA response to cocaine	Gentile *et al.*, 2018
		Hcrt Knockdown	Attenuates self administration in proggressive ratio	Schmeichel *et al.*, 2017
		Hcrt Knockout	Blunted intake at highest dose and reduced response to drug after abstinence	Steiner *et al.*, 2018
	EtOH	SB-334867	Reduced EtOH intake and cue induced reinstatement in EtOH preferring rats	Moorman, James, Kilroy, & Aston-Jones, 2017
		GSK1059865	Reduced EtOH vapor induced EtOH drinking in dependent mice	Lopez, Moorman, Aston-Jones, Becker, 2016
		TCS-OX2-29	Anterior PVT injections of OX _2_R antagonist reduces EtOH intake. EtOH consumption increases OX _2_R mRNA in PVT	Barson, Tin Ho, Leibowitz, 2015
		/	In a white population, OX _2_R polymorphism was associated with rate of alcohol dependence independent of age or gender	Klepp *et al.*, 2017
		/	Context induced reinstatement associated with various levels of Hcrt neuron activity across the LH	Moorman, James, Kilroy, & Aston-Jones, 2016
		/	Voluntary EtOH drinking in zebrafish increases Hcrt expression in hypothalamus	Sterling, Karatayev, Chang, Algava, & Lebowitz, 2015
	Opioids	SB-334867	Differentially modulates hedonic and motivational effects of remifentanyl in high and low takers	Porter-Stransky, Bentzley, & Aston-Jones, 2017
		SB-334867	Intra-VTA inections attenuate morphine CPP	Farahimanesh, Zarrabian, & Haghparast, 2017
		SB-334867	Intra-DG injection attenuates drug induced reinstatement of morphine CPP	Ebrahimian *et al.*, 2016
		TCS-OX2-29	Intra-VTA injections attenuates morphine CPP	Farahimanesh, Zarrabian, & Haghparast, 2017
		TCS-OX2-29	Intra-DG injection attenuates drug induced reinstatement of morphine CPP	Ebrahimian *et al.*, 2016
		NBI-80713	Reduced heroin self administration in long access paradigm and increase in OX _2_R mRNA in the AMY	Schmeichel *et al.*, 2015
		/	Morphine CPP increases Hcrt1 release in DG	Guo *et al.*, 2016

### Stress and anxiety

Hcrt plays a role in the coordination of stress responses. Plasticity in the Hcrt system is thought to contribute to long-term dysregulation of arousal seen in certain psychiatric disorders ^85, 86^. This may be an adaptive response to repeated stress, where heightened arousal and vigilance are needed under conditions of instability or high threat ^87^. Recent literature has supported the idea that activation of OX _1_R promotes anxiety-like behavior. For example, in rodent models of panic, an extreme form of anxiety, animals with panic vulnerability treated with the OX _1_R antagonist compound 56 reduced panic-like behaviors in a sodium lactate model of panic induction ^88^. Similarly, treatment with the OX _1_R antagonist JNJ-54717793 attenuates panic-like behavior and cardiovascular responses in both the sodium lactate model of panic and a carbon dioxide (CO _2_) model of panic provocation ^89^. Additional studies within the CO _2_ model that screened selective Hcrt receptor antagonists (SORAs) and dual Hcrt receptor antagonists (DORAs) found that both a SORA1 (compound 56) and a DORA-12 attenuate anxiety-like behaviors but that a SORA2 did not ^90^. Importantly, these data provide a promising treatment route, as animals treated with SORA1 and DORA-12 showed no significant changes in sleep ^90^. Currently, the levels of benzodiazepines needed to achieve anxiolytic effects are also sedating; as discussed here, OX _1_R antagonists can have anxiolytic effects without impacting sleep ^90^.

Although the mechanism of action of the wake-promoting drug modafinil is mainly through activation of DA circuitry, it also activates Hcrt neurons and is used for the treatment of narcolepsy. Treatment with modafinil after a traumatic experience reduces the incidence of post-traumatic stress disorder (PTSD), a disorder characterized by anxiety and hyperarousal. The anxiolytic effect of this treatment may be due to its interference with normal sleep-dependent memory processes ^91^. However, the benefits of modafinil treatment may go beyond this, as it has been shown to stimulate adaptive stress responses in an animal model of PTSD ^92, 93^. In a model of orofacial pain-induced anxiety, rats given injections of capsaicin into the upper lip showed increased anxiety-like responses on the elevated plus maze. Administration of Hcrt exacerbates this response while treatment with OX _1_R antagonists inhibits orofacial pain-associated anxiety ^94^. In another study, differential effects of OX _1_R antagonism were observed. The OX _1_R antagonist SB-334867 influenced arousal (mobility/immobility in an open field) but not anxiety-like behavior (center exploration) in conditions of mild stress in male rats ^95^. Yet Hcrt knockout mice show increased anxiety in the open-field test, light-dark box test, and predator scent avoidance test despite intact fear learning ^96^. Likewise, OX _1_R receptor knockout mice show increased anxiety and reduced social interaction, increased startle responses, and altered depressive-like behavior ^97^. Although genetic knockout results do not completely contradict findings from pharmacological studies, they do showcase the necessity to use the newest genetic techniques to parse out the role of Hcrt in anxiety. Two points must be made with regard to these findings: first, knockout models may result in compensatory mechanisms that may explain how Hcrt-null or OX _1_R-deficient mice display lower anxiety. Second, models of stress discussed here vary greatly, and the conclusions drawn from these works may reflect the differences in the circuits underlying different types of anxiety. Thus, findings must be interpreted within the context of pharmacological, genetic, and behavioral manipulations used in these studies.

Recent work is also characterizing how individual differences in baseline Hcrt activity may pose resilience or susceptibility to stress. Rats that show low expression of preprohypocretin mRNA are resilient to social stress, and further manipulations show that chemogenetic inhibition of Hcrt reduces depressive-like behavior in otherwise stress-susceptible rats ^98^. Together, these data suggest that the activity of Hcrt on stress may be context or stressor specific but additionally that individual differences at baseline may influence stress resilience.

### Motivation and addiction

The mesolimbic DA system, which originates in the VTA and projects to the striatum, is a key region for the processing of reward and reinforcement ^99, 100^. These processes necessitate and evoke arousal states to monitor reinforcers and facilitate learning ^101^. Reciprocally, motivational states impact arousal so as to facilitate the seeking of rewards and the avoidance of punishments ^102, 103^. As discussed above, LH-Hcrt neurons send excitatory projections to the VTA ^13, 50, 51^. Thus, the VTA may be an optimal region by which Hcrt can influence motivated arousal states. The majority of recent advances made in this field have investigated the effects of Hcrt manipulations on motivation for cocaine and ethanol (EtOH). To date, these studies suggest that Hcrt1 plays a role in motivation for drug reward, especially when drug presentation is dependent on effortful responses on the part of the animal. Here, we discuss the role of Hcrt in addiction and motivation, focusing on cocaine, alcohol, and opioids.

Hcrt knockdown attenuates cocaine self-administration under progressive ratio schedule (that is, Hcrt knockdown lowers cocaine breakpoint) but not under a fixed ratio schedule ^104^. Similarly, Hcrt-deficient mice show reduced cue-induced cocaine-seeking behavior following a period of abstinence, suggesting a role for Hcrt in relapse behavior ^105^. Additionally, these animals show blunted cocaine intake at the highest dose and reduced behavioral responses to cocaine after abstinence ^105^. Additional work from Navarro and colleagues further supports the role of Hcrt in relapse behavior ^106^. In particular, their work shows that cocaine acts at and alters activity of corticotropin-releasing factor receptor (CRF _1_R)/OX _1_R heterodimers within the VTA. Action of cocaine at these sites disrupts Hcrt/CRF crosstalk even 24 hours after a single systemic injection and may be a mechanism underlying stress-induced cocaine relapse ^106^.

Indeed, Hcrt may play a unique role in cue-reward associations, as OX _1_R antagonism via SB-334867 only decreases cocaine demand in the presence of cues. SB-334867 treatment also blocks cue-induced reinstatement of drug seeking—an effect most pronounced in high-demand animals (animals with the greatest cue-dependent behavior). This suggests that OX _1_R increases the reinforcing efficacy of cocaine-associated cues but not of cocaine alone. This supports the notion that Hcrt plays a role in the ability of conditioned cues to elicit motivational responses ^107^. Recent *in vivo* measurements of DA activity are beginning to inform the mechanisms that may underlie these observed effects on cocaine reinforcement. For example, Hcrt knockdown within the VTA delays acquisition of cocaine self-administration and reduces motivation for cocaine under a progressive ratio schedule while reducing DA release in the ventral striatum, DA uptake, and cocaine-induced DA reuptake inhibition at striatal terminals ^108^. Similarly, OX _1_R blockade with RTIOX-276 attenuates motivation for cocaine and reduces the number of DA transients, DA release evoked by cocaine cues, and cocaine-induced DA reuptake inhibition as measured by fast scan cyclic voltammetry (FSCV) ^109^. Suvorexant, a DORA, attenuates the motivational properties of cocaine as measured by progressive ratio and place conditioning. Additionally, treatment with Suvorexant also reduces the hedonic properties of cocaine as measured by ultrasonic vocalizations. Additionally, DORA treatment reduced cocaine-induced elevations in ventral striatal DA ^110^. Work by Prince and colleagues suggests that effects of the DORA may be mediated by OX _1_R, as blockade of OX _2_R receptors alone has no effect on DA signaling or self-administration of cocaine ^111^. However, blocking of OX _1_R or both OX _1_R and OX _2_R decreases motivation for cocaine as measured by self-administration under a progressive ratio schedule and reduces the effects of cocaine on DA signaling as measured by FSCV ^111^.

In the case of EtOH, Hcrt antagonism generally reduces EtOH consumption. In a voluntary EtOH intake model in zebrafish, it was seen that intake of EtOH increases Hcrt expression in the hypothalamus ^112^. OX _1_R antagonism with SB-334867 reduces EtOH self-administration in alcohol-preferring rats ^113^. Similarly, the OX _1_R antagonist GSK1059865 reduces EtOH drinking in EtOH-dependent mice ^114^. In a model of EtOH seeking and preference, activation of the LH is correlated with degree of seeking in context-induced reinstatement and degree of preference in home cage EtOH preference testing. Interestingly, cue-evoked reinstatement shows no correlation with Hcrt activation in any region. This suggests that there is a relationship between Hcrt activity in the LH and EtOH seeking and preference behavior but that cue-induced reinstatement for alcohol may be mediated by a different mechanism ^115^. Interestingly, EtOH consumption increases OX _2_R mRNA within the anterior paraventricular nucleus of the thalamus and local antagonism of OX _2_R reduces total EtOH intake ^116^.

The interactions of Hcrt with opioid rewards are particularly interesting, as the endogenous opioid dynorphin (Dyn) is expressed in 94% of Hcrt neurons and Hcrt and Dyn are thought to be co-released at Hcrt terminals within the VTA ^117^. The interactions of these neurotransmitters are beyond the scope of this review; however, of major relevance is the point that these neurotransmitters have opposing yet complementary actions on VTA cellular excitability ^117– 121^. OX _1_R antagonism with SB-332867 modulates demand for the opioid drug remifentanil in low takers but not in high takers ^122^. Additionally, intra-VTA injections of the OX _1_R antagonist SB-334867 attenuate morphine conditioned place preference (CPP) acquisition and expression. Interestingly, in the case of opioid reward, OX _2_R antagonism via TCS-OX2-29 also significantly attenuates morphine CPP acquisition and expression, suggesting that both receptors within the VTA are important for expression of morphine reward ^123^. Similarly, systemic treatment with the OX _2_R antagonist NBI-80713 dose-dependently reduces heroin self-administration in a long-access paradigm. Long-access heroin self-administration paradigms are thought to mimic compulsive drug taking; thus, OX _2_R antagonism may be particularly effective at influencing drug-associated compulsivity. Similar effects have been observed in the hippocampal dentate gyrus (DG), which receives Hcrt projections from the LH and interacts with the VTA to play an important role in the linking of drug reward with contextual cues ^124^. In a stress- and drug-induced model of morphine reinstatement, intra-DG administration of OX _1_R and OX _2_R antagonists attenuates drug priming-induced reinstatement dose-dependently with no effect on stress-induced reinstatement ^125^. Similarly, morphine CPP increases Hcrt1 release in the DG and OX _1_R antagonism via SB-334867 ameliorates morphine CPP. These findings suggest that Hcrt actions at the DG may influence the learning of drug-context associations ^126^.

Finally, additional work has begun to delineate the effect of Hcrt on motivation at VTA terminal sites such as the nucleus accumbens (NAc) ^127^. Blomeley and colleagues used optogenetics and electrophysiology to characterize a direct Hcrt→DA D _2_ excitatory circuit that is necessary for the expression of risk avoidance behavior in mice ^127^. Indeed, increased DA D _2_ neuron activation caused animals to avoid risks such as crossing a predator-scented chamber to attain a food reward and chemogenetic silencing of accumbal DA D _2_ cells inhibited Hcrt-mediated avoidance. Importantly, these data showcase how Hcrt can influence adaptive behavioral inhibition even in the presence of rewards. These data open up new opportunities of research, such as characterizing the effects of Hcrt on different subregions of the NAc, which is a heterogeneous structure with distinct electrophysiological properties ^128, 129^. Additional lines of research should investigate how Hcrt-mediated motivation in the NAc is impacted by diurnal rhythms as well as sleep disturbance and how the Dyn system interacts in this region to modulate motivation ^120, 130^.

### Cognitive function and memory

Studies suggest that Hcrt deficiency is associated with memory deficits. Hcrt deficiencies negatively impact working memory as tested in a non-matching-to-place T-maze task ^131^. Hcrt/ataxin-3 transgenic mice (a progressive model of narcolepsy), which become Hcrt deficient at 12 weeks old, show impaired avoidance memory in a two-way active avoidance paradigm in which an animal has to perform a specific motor response to avoid an aversive stimulus. Hcrt1 administration reverses memory deficits, suggesting that Hcrt plays a role in hippocampal-dependent consolidation of two-way active avoidance memory ^132^. Chemogenetic activation of Hcrt neurons improves short-term memory for novel locations, a function that putatively supports foraging and exploration ^133^.

Pain negatively influences memory processing in ways that may be influenced by Hcrt. In the Morris water maze (MWM) (a test of spatial learning and memory), orofacial pain-induced memory impairments are exacerbated by the OX _1_R antagonist SB-334867 whereas administration of Hcrt1 prevented these spatial memory deficits ^134^. Importantly, injections were directed at the trigeminal nucleus caudalis, which is a central relay for orofacial pain. Thus, the observed effect on memory may be via alterations in the experience of pain itself rather than the formation of a pain-associated memory ^134^. In a similar study by Raoof and colleagues, orofacial pain memory was mediated by Hcrt at the level of the hippocampus (HPC). Intra-hippocampal injections of Hcrt1 inhibit pain-induced memory impairments as measured by the MWM. However, treatment with the OX _1_R antagonist SB-334867 had no effect on learning and memory ^135^. Indeed, the HPC is a critical region for memory function and Hcrt action at this site may influence memory processes via its influence on the induction of long-term potentiation (LTP). *In vitro* studies show that OX _1_R antagonists significantly decrease the firing rates of hippocampal CA1 neurons, showing that the effect of Hcrt on these neurons is excitatory ^136^. Additional *in vitro* electrophysiology studies demonstrate that Hcrt1 may bidirectionally modulate HPC CA1 function. Specifically, moderate doses of Hcrt1 inhibit LTP while subnanomolar concentrations result in re-potentiation via OX _1_R and OX _2_R ^137^. It is important to note that the Hcrt manipulations discussed here may have influenced sleep and therefore resulting memory problems may be sleep dependent and thus only indirectly dependent on Hcrt.

## Part III: quantitative modeling of hypocretin circuits

Computational modeling of the Hcrt network remains a relatively unexplored frontier. Development of analytical models of Hcrt function will inform our interpretation of data gathered through empirical study and drive the development of testable hypotheses. In particular, computational modeling of Hcrt networks will prove essential for our understanding of the following three questions: (1) how do internal or external physiological states influence arousal? (2) How does the heterogeneity of the system (that is, genetic, afferent, and efferent diversity) contribute to network dynamics? (3) How does Hcrt function as a volume transmitter to produce both generalized and specific effects? Ultimately, integration of these models with experimental approaches will allow for understanding of the network as a whole as well as monosynaptic interactions.

### Models of hypocretin network in arousal

Current models have described Hcrt as functioning within a “flip/flop” model where it stabilizes wakefulness, preventing aberrant switches between mutually exclusive states ^138^. This model, however, cannot account for overlapping states of arousal such as those observed in narcolepsy or RBD in which REM sleep can co-occur with conscious awareness ^139, 140^. Additionally, this model does not factor in the many systems that interact to influence arousal. These observations make it necessary to revise the binary nature of the flip/flop model. Studies have expanded the model by characterizing a circuit with hierarchical gating of additional neural circuits, feedback, and redundancy ^141^. This hierarchical model provides a framework on which to add motivational influences on arousal states. Indeed, animals can adapt their sleep on the basis of internal and external variables such as migration or predator avoidance or to increase the likelihood of mating ^142– 144^. Recently, an alternative has been proposed in which sleep-to-wake transitions are predicted on the basis of inputs with different “weights” onto an integrator neuron ^145^. An integrator neuron would continuously compute probabilities of wakefulness on the basis of functional connectivity of the system as well as physiological factors such as stress or circadian phase. Diversity of neuronal responses to stimuli can be integrated within this model to account for the heterogeneity of the system. In this vein, Schöne and Burdakov acknowledge the necessity of an adaptive behavioral control system that can respond to unpredictable changes in the environment ^146^. Thus, they propose a model of brain arousal control modules organized in a feedback loop by which Hcrt can gate relevant information on the basis of environmental and homeostatic needs ^146^. We look forward to the future advancement of this area of Hcrt research that will undoubtedly expand our understanding as an adaptable regulator of arousal.

### Volume transmission

Volume transmission (VT) is a mechanism of neural signaling by which neurotransmitters can exert actions on cells in close proximity as well as distant targets. In VT, neurotransmitters signal via diffusion within extracellular fluid ^147, 148^. This type of release is thought to allow for modulation of neural activity via long time courses and greater distances ^147– 149^. VT may happen via cellular pores, diffusion through the plasma membrane, exocytosis, or reversal of transporter proteins ^149^. To date, actions of Hcrt at the dorsal lateral geniculate nucleus (DLG) and the DRN (aside from already-known synaptic actions) have been theorized to be exerted via VT ^150, 151^. Observations of Hcrt1 immunoreactivity in many non-synaptic varicosities located far from synapses with axons forming asymmetric synapses suggest that DRN excitation via Hcrt1 may be via this mechanism ^150^. Indeed, the DRN plays an important role in the regulation of arousal and both synaptic and VT mechanisms may support long-term cortical arousal ^25, 36^. In a separate set of findings, Hcrt was found to powerfully modulate neurons of the DLG despite only sparse expression of Hcrt nerve terminals in the region, suggesting that these actions are via VT ^151^. Additionally, a recent study of melanin-concentrating hormone (MCH), a hypothalamic peptide important for the regulation of feeding, shows that MCH neurons project to ventricular regions where they increase MCH levels in the cerebrospinal fluid (CSF) and stimulate feeding ^152^. MCH neurons are intermingled with Hcrt neurons in the LH, and the authors measure that 40% of Hcrt neurons also project to the CSF where they are poised to signal via VT to influence distal targets ^152^. Further investigations should determine whether Hcrt acts via VT and, if so, how its activity is influenced by (1) temporal and spatial release dynamics, (2) diffusion and dilution parameters, and (3) transporter kinetics in order to characterize its effective radius.

## Future directions and conclusions

As reviewed here, the ever-growing database on Hcrt continues to broaden our conceptualization of these peptides as more than just regulators of sleep-to-wake transitions. Technical advances have allowed ever more precise measurement and manipulation of these circuits which will continue to inform our understanding of this circuit. To date, therapeutic advances have allowed the effective targeting of Hcrt circuitry for the treatment of narcolepsy and insomnia, and research discussed here provides evidence for the potential of this system for the treatment of anxiety, addiction, and memory deficits. Integration of these findings with analytical models will provide a novel means for explaining and interpreting biological observations so as to gain a holistic understanding of their role in physiology and behavior.
